# The Effect of Alcohol on Postprandial and Fasting Triglycerides

**DOI:** 10.1155/2012/862504

**Published:** 2011-09-22

**Authors:** Albert Van de Wiel

**Affiliations:** Department of Internal Medicine, Meander Medical Center, Utrechtseweg 160, 3818 ES Amersfoort, The Netherlands

## Abstract

Alcohol has a significant additive effect on the postprandial triglyceride peak when it accompanies a meal containing fat, especially saturated fat. This results from a decrease in the breakdown of chylomicrons and VLDL remnants due to an acute inhibitory effect of alcohol on lipoprotein lipase activity. Furthermore, alcohol increases the synthesis of large VLDL particles in the liver, which is the main source of triglycerides in the hypertriglyceridemia associated with chronic excessive alcohol intake. In case of chronic consumption, lipoprotein lipase activity seems to adapt itself. The effect of alcohol on adipose tissues is less clear. Sometimes, a severe hypertriglyceridemia induced by alcohol (SHIBA) can be observed, especially in patients with type 2 diabetes mellitus and/or obesity increasing the risk of pancreatitis.

## 1. Introduction

Hypertriglyceridemia (HT) may result from genetically determined disturbances in lipid metabolism but may also be secondary to conditions such as obesity, diabetes mellitus, hypothyroidism, the nephrotic syndrome, and the use of medication. Quite a number of drugs can be associated with HT including *β*-blockers, steroids, diuretics, oestrogens, immunosuppressants, cytostatics, and antiviral drugs [1]. Although its health effects are continuously a subject of debate, alcohol (ethanol) can be added to this list [2, 3]. 

Since both food and alcohol are known to affect lipid metabolism, a number of studies have been directed to their combined effect. This paper will focus on triglycerides after the consumption of such mixed meals as well as on the observation that alcohol may induce a very severe form of HT, which may be of clinical relevance with an increased risk of pancreatitis [4].

## 2. Alcohol and Triglycerides in Animal Studies

The metabolic effects of alcohol on the liver and lipid metabolism are known for many years and have been extensively studied by Lieber and many of his coworkers [5]. Although the administration of one dose of a diet containing alcohol (3 g/kg) given to rats produces a significant increase in mesenterial lymph flow, lipid output, and incorporation of dietary fat into lymph lipids, this does not result in HT [6]. By contrast, previous feeding of alcohol for several weeks results in postprandial hyperlipemia after a single dose of the mixed meal and even after such a dose without the alcohol component. Baboons fed with a liquid diet containing 50% of energy as ethanol for 5–16 months develop HT and alcoholic liver injury (fatty liver) [7]. This HT results from an increased production of large VLDL particles by the liver, while the splanchnic extraction of TG from chylomicron- and VLDL-remnants is secondarily enhanced. In another rat experiment, Daher et al. confirmed a rise in plasma triacylglycerol and chylomicron concentrations after ethanol ingestion but also found a decreased chylomicron size with a change in cholesterol and phospholipid content indicating enhanced liver bile secretion [8]. In case the animals were put on a moderate alcohol diet for a period of ten weeks, their postprandial HT and hyperchylomicronemia were less pronounced. This is probably related to an adaptive increase of lipoprotein lipase (LPL) activity in case of chronic moderate alcohol consumption [9]. In contrast, an acute ingestion of ethanol lowers LPL activity. Another mechanism which may contribute to postprandial HT is a decrease of lipogenesis and glucose oxidation in adipose tissue, as shown in rats after chronic ethanol feeding [10]. Kang et al. [11] studied triglyceride turnover in white adipose tissue and showed that chronic alcohol consumption inhibits the antilipolytic action of insulin. It has also been shown that the lipemic response to alcohol is related to the stage of liver disease, since in cirrhosis, in contrast to steatosis, fasting lipid response is neglectable, but postmeal chylomicron response is increased [12].

Apart from serum HT, alcohol may also induce accumulation of triacylglycerols in the liver, leading to steatosis hepatis. This influence of alcohol can be partly explained by impairment of AMP-activated protein kinase (AMPK), which enzyme plays a central role in hepatic fatty acid metabolism [13]. Several dietary regimes have proven to be able to influence this process of alcohol-induced fatty liver including medium-chain triglycerides and fish oil [14, 15].

Interestingly, the consumption of alcohol stimulates the intake of fat, while dietary fats stimulate the consumption of alcohol, a vicious cycle probably mediated by hypothalamic peptides [16].

## 3. Triglycerides after Mixed Meals in Humans

When normal healthy volunteers consume wine, in total 30 g of alcohol, during a standard diner, postprandial TG, measured one hour after intake, increase by 15.3%, but after overnight fasting, values have returned to normal [17]. This effect is clearly related to alcohol and not to the type of drink, since in a similar experiment, no differences were found between wine, beer, and spirits [18]. In another experiment of these investigators, they showed that TG levels reach their peak three hours after dinner with the same response in men and women [19]. Even so, the response is not different in men with a low-risk profile for cardiovascular disease compared to men with a high-risk profile [20].

Since both alcohol and exercise have an effect on postprandial lipemia and TG clearance, their combined effect has been studied. El-Sayed and Al-Bayatti [21] studied plasma TG concentrations after exercise followed by a diet with and without alcohol. In the control trial, when subjects consumed a standardized lunch after their 35 minutes of exercise (VO2max 70%), TG showed no significant change. However, when alcohol was consumed with the lunch, TG concentration increased substantially 5 hours during recovery. The mechanism responsible for this TG rise was not studied, but inhibition of LPL activity by alcohol may play a role. On the other hand, Hartung et al. were able to show that alcohol-induced increases in postprandial lipemia and retardation of TG clearance occur in inactive men but not in exercise-trained subjects [22].

Even if the diet contains a lot of fat resulting in high postprandial TG levels, the addition of alcohol still has a significant additive effect. Franceschini et al. [23] performed an experiment in which normolipemic subjects either consumed 70 g of fat or this amount of fat in combination with 40 g of ethanol. Four to six hours, after the fat intake TG levels rose by 70% but after the intake of both fat and ethanol by 180%. A similar study was designed by Pownall [24] in which three different fat loads were given to normal subjects with and without alcohol. The fat loads consisted of saturated fat, polyunsaturated fat, or polyunsaturated fat with omega-3 fatty acids. Preprandial alcohol increased postprandial lipemia, an effect that was most profound with the saturated fat load. Alcohol had no effect on the plasma concentrations of free fatty acids derived from peripheral tissue but appeared to decrease the plasma concentration of free fatty acids from dietary origin. These data are highly suggestive for an impairment of chylomicron hydrolysis due to inhibition of LPL. Fielding et al. [25] also found no arguments for an effect of alcohol on the release of nonesterified fatty acid into the circulation. 

Because of the increased risk of cardiovascular diseases in diabetes patients and the possible cardioprotective effect of alcohol, Dalgaard et al. [26] studied the effect of a mixed meal on postprandial lipemia and incretin levels in type 2 diabetes patients. Early in the postprandial phase, alcohol suppresses the incretin responses and increases the late postprandial TG levels. This alcohol-induced suppression of the incretin response resulting in lower insulin levels may contribute to the impaired TG clearance in type 2 diabetes patients but may also be operative in nondiabetics.

## 4. Fasting Triglycerides and Regular Alcohol Consumption

In contrast to moderate alcohol consumption, excessive intake may cause HT even in the fasting state [5, 17]. This effect seems to be more pronounced in African-Americans than in white Americans [27]. When regular and binge drinkers cut down their alcohol intake, a more or less similar drop (0.22 and 0.26 mmol/L, resp.) in fasting TG is observed, indicating that HT is more related to the amount of alcohol consumed than to the pattern of drinking [28]. Pownall et al. [29] studied the effect of the consumption of two alcoholic drinks in the fasting state in patients with mild hypertriglyceridemia (2.3–8.5 mmol/L) in comparison with normal lipemic individuals. At six hours (peak), TG concentration increased only 3% in the HT group and 53% in the nonhypertriglyceridemic group. They conclude that alcohol intake alone in not an important determinant of plasma TG concentration in individuals with HT. 

On the other hand in a recent study analyzing the underlying disorders in patients with severe HT, alcohol proved to be of dominant importance [4]. In 300 patients with TG levels exceeding 11.3 mmol/L (1000 mg/dL), excessive alcohol was present in almost a quarter of all patients and in even 43% of the highest quartile of TG levels. Especially, patients with the combination of alcohol abuse, diabetes mellitus, and obesity, for which the authors introduced the term SHIBA syndrome (severe hypertriglyceridemia influenced by alcohol), are prone to develop extremely high TG levels. In those cases, there is an increased risk of developing pancreatitis. Both the effects of excessive alcohol intake and the lack of insulin or insulin resistance push TG metabolism in the same direction.

## 5. Conclusions

The consumption of alcohol-containing drinks has become an accepted part of lifestyle in most societies. The health effect of alcohol, however, has always been subjected to debate. Moderate alcohol consumption is associated with a lower risk of cardiovascular disorders, and the pattern and amount of alcohol are of more importance than the type of alcoholic beverage [2, 30]. One of the underlying mechanisms for this beneficial effect is its influence on lipids especially the increase in plasma HDL-cholesterol [31]. In case of moderate drinking, 1–3 glasses a day for men and 1-2 glasses for women, hardly any effect is seen on triglycerides.

However, excessive alcohol intake may cause hypertriglyceridemia not only postprandially, but also in the fasting state. This is mainly due to an increase in the synthesis of large VLDL particles in the liver. When alcohol consumption is accompanied by a meal containing fat, especially saturated fat, it has a significant additive effect on the postprandial triglyceride peak. This peak is for the most part the result of a retardation of chylomicron breakdown and to some extent of that of VLDL remnants. Most likely, this should be attributed to an inhibition of lipoprotein lipase activity by alcohol. In case of moderate and regular alcohol intake, adaptation restores LPL activity.

In some cases, alcohol may be responsible for extremely high levels of triglycerides with an increased risk of pancreatitis. Especially, patients with the metabolic syndrome seem prone to develop such a severe hypertriglyceridemia.

Whether these changes in both postprandial and fasting triglycerides have clinical implications for cardiovascular disorders needs further exploration.
